# Characterization of the relationship between polar and lateral flagellar structural genes in the deep-sea bacterium *Shewanella piezotolerans* WP3

**DOI:** 10.1038/srep39758

**Published:** 2016-12-22

**Authors:** Huahua Jian, Han Wang, Xianping Zeng, Lei Xiong, Fengping Wang, Xiang Xiao

**Affiliations:** 1 State Key Laboratory of Microbial Metabolism, School of Life Sciences and Biotechnology, Shanghai Jiao Tong University, Shanghai, PR China; 2 State Key Laboratory of Ocean Engineering, School of Naval Architecture, Ocean and Civil Engineering, Shanghai Jiao Tong University, Shanghai, PR China

## Abstract

Bacteria with a dual flagellar system, which consists of a polar flagellum (PF) and several lateral flagella (LF), have been identified in diverse environments. Nevertheless, whether and how these two flagellar systems interact with each other is largely unknown. In the present study, the relationship between the structural genes for the PF and LF of the deep-sea bacterium *Shewanella piezotolerans* WP3 was investigated by genetic, phenotypic and phylogenetic analyses. The mutation of PF genes induced the expression of LF genes and the production of LF in liquid medium, while the defective LF genes led to a decrease in PF gene transcription. However, the level of PF flagellin remained unchanged in LF gene mutants. Further investigation showed that the *flgH2* gene (encoding LF L-ring protein) can compensate for mutations of the *flgH1* gene (encoding PF L-ring protein), but this compensation does not occur between the flagellar hook-filament junction proteins (FlgL1, FlgL2). Swarming motility was shown to specifically require LF genes, and PF genes cannot substitute for the LF genes in the lateral flagella synthesis. Considering the importance of flagella-dependent motility for bacterial survival in the abyssal sediment, our study thus provided a better understanding of the adaptation strategy of benthic bacteria.

Flagella are the key organelles for bacterial locomotion and facilitate movement towards favourable conditions or away from detrimental environments^1^,^2^. Moreover, in various bacteria, such as *Pseudomonas aeruginosa* and *Vibrio cholerae*, flagella were shown to be involved in biofilm formation^3^. Although the number and distribution of flagella on the cell surface of bacteria varies among the different species, most flagella can be classified into two types, polar and lateral, depending on the arrangement of the flagella. Many bacteria such as *P. aeruginosa* and *V. cholerae,* possess a single polar flagellum (PF). However, some microorganisms can express multiple lateral flagella (LF), such as, *Escherichia coli, Salmonella enterica* and *Proteus mirabilis*^4^. Interestingly, a number of species, such as *V. parahaemolyticus* and *Aeromonas hydrophila*, have been shown to harbour dual flagellar systems^5^. In *V. parahaemolyticus*, the Na^+^-powered PF is used for swimming in liquid environments, and the H^+^-powered LF are utilized for swarming on solid surfaces or under high-viscosity conditions^5^. The PF is produced continuously, while an inducible flagellar system synthesizes LF under conditions that inhibit PF function^6^. As shown in *V. parahaemolyticus*, the PF and LF systems have several distinguishing characteristics and are under the control of independent regulatory hierarchies: the master regulators controlling PF and LF gene expression were reported as the σ^54^-dependent FlaK and the transcriptional activator LafK, respectively^5^,^6^.

The motility and dual flagellar systems of the deep-sea bacterium *Photobacterium profundum* SS9 have been investigated and the gene mutants for the PF were shown to have impaired motility under all tested conditions; furthermore, the motility of the LF gene mutants was only defective under conditions of high pressure and high viscosity^7^. Moreover, the expression level of the LF gene in *P. profundum* SS9 was significantly induced by elevated pressure and increased viscosity^7^. A dual flagellar system has also been identified in *Shewanella piezotolerans* WP3 (here referred to as WP3), which was isolated from West Pacific sediment at a depth of 1914 m^8^,^9^. Our previous study revealed that the expression of the two flagellar systems is differentially regulated at low temperature and high pressure and that the LF system was found to be important for WP3 growth at 4 °C^9^, suggesting that the flagellar system is significantly involved in benthic environmental adaptation.

Interactions between the PF and LF systems have been elucidated in the well-studied dual flagellar system of *V. parahaemolyticus*. Mutations affecting PF performance resulted in the production of LF in liquid medium^10^. Moreover, the LF regulator LafK could influence the expression of PF genes by compensatory activation^11^. Nevertheless, the relationship between the polar and lateral flagellar structural genes is still largely unknown. Specifically, it remains to be investigated whether the components of the dual flagellar system influence each other and whether they are interchangeable, although they may share some common regulators. In this study, the relationship between the PF and LF genes of WP3 was characterized by genetic and phenotypic analyses. The mutation of PF genes induced the expression of LF genes and LF production in liquid medium, while defective LF genes resulted in the decrease of PF gene transcription. Intriguingly, an LF L-ring protein FlgH2 could partially compensate the function of the PF protein FlgH1, thus the bacterium retained the ability to produce a PF and maintained their swimming motility. These findings contribute to a comprehensive understanding of the relationship between the dual flagellar systems in deep-sea bacteria.

## Results

### The swimming motility of WP3 was decreased but not completely lost by impairment of the PF and LF genes

**Figure 1:**
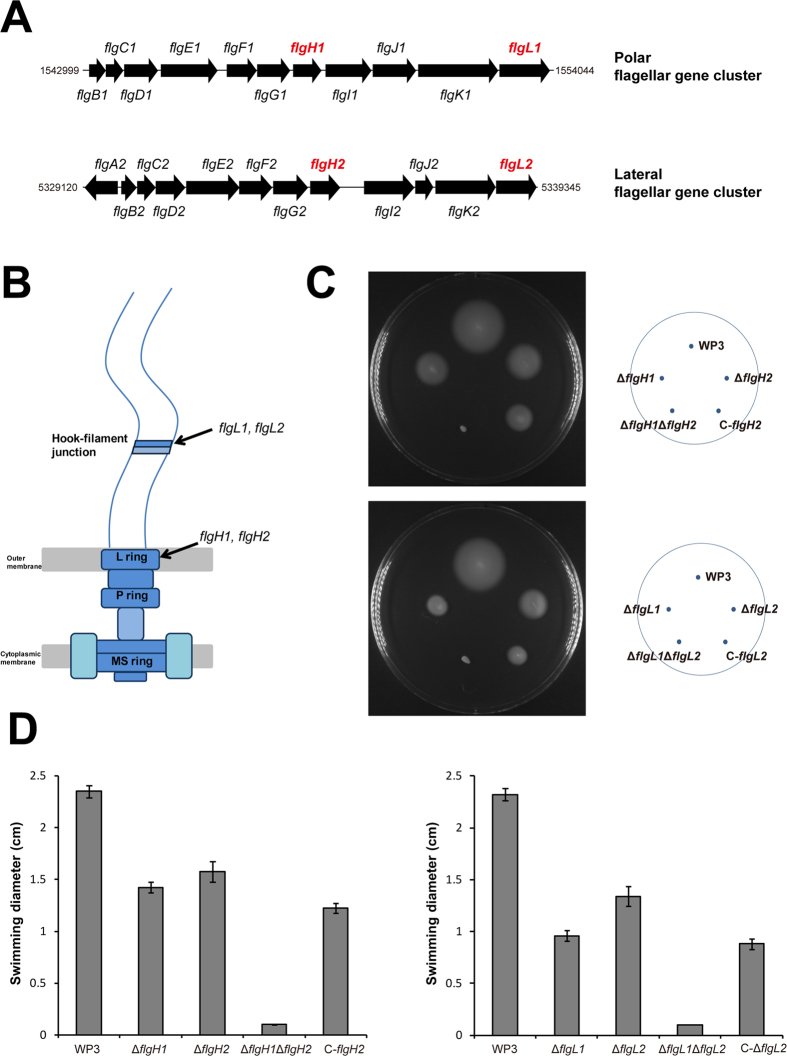
Characterization of the swimming motility of WP3 flagellar gene mutants. (**A**) A part of the flagellar gene cluster of WP3 is shown. The genomic locations of the two gene clusters are indicated, and the genes that were selected for mutational analysis are shown in red. (**B**) Schematic of the flagellum structure showing the deleted genes encoding flagellar components. (**C** and **D**) Swimming motility assays of the flagellar gene deletion mutants. The results shown are representative of at least three independent experimentsand the error bars indicate the standard deviations.

In our previous study, the swimming motility of WP3 was shown to be decreased, but not completely lost by the deletion of either a PF gene (*swp1508, flgL1*) or an LF gene (*swp5125, motA2*)^9^. To confirm this phenotype, two pairs of genes, *flgH1*/*flgH2* and *flgL1*/*flgL2,* which encode the L-ring protein and the hook-filament junction protein of the WP3 flagellum (Fig. 1A and 1B), respectively, were selected for the mutational analysis. Four single-gene deletion mutants Δ*flgH1,* Δ*flgH2,* Δ*flgL1* and Δ*flgL2* were constructed and swimming motility was assessed. The swimming motility of Δ*flgH1 and* Δ*flgL1* was decreased but not completely eliminated (Fig. 1C and 1D). Interestingly, the deletion of the LF genes *flgH2* and *flgL2* also led to a significant decrease in swimming motility.

To further confirm that the flagellar genes are responsible for the swimming motility, the double deletion mutants Δ*flgH1*Δ*flgH2 and* Δ*flgL1*Δ*flgL2* were constructed. As expected, swimming motility was completely eliminated. To verify the involvement of LF genes in the swimming motility of WP3, the intact *flgH2* and *flgL2* genes were introduced into Δ*flgH1*Δ*flgH2* and Δ*flgL1*Δ*flgL2*, respectively. The motility assay showed that the swimming motility partially recovered, which is similar to the results with Δ*flgH1* and Δ*flgL1* (Fig. 1C and 1D). As a growth assay demonstrated that there is no significant difference between wild-type WP3 and the flagellar gene mutants (Supplementary Fig. S1), the change in motility is not attributed to a growth deficiency. Taken together, these results indicated that the swimming motility of WP3 was decreased but not completely lost by impairment of either the PF or LF genes, suggesting that there is functional substitution of the homologous genes between the PF and LF system, thus influencing the swimming motility of WP3.

### LF was induced by the mutation of PF genes in liquid environment

**Figure 2:**
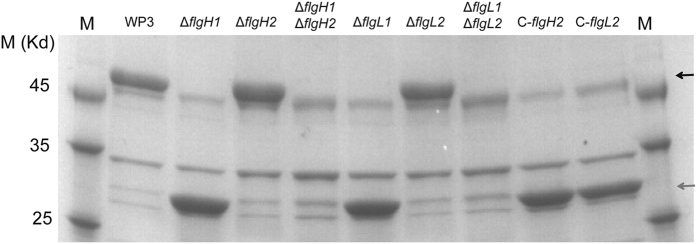
SDS-PAGE analysis of isolated flagellins from WP3 flagellar gene mutants and complemented strains. Flagellins from the same volume of log-phase cultures adjusted to the same optical density were extracted and separated using SDS-PAGE. The black and grey arrows indicate the bands representing the polar and lateral flagellins, respectively. The full-length gels are presented in Supplementary Figure S4.

In the wild-type WP3 strain, the polar and lateral flagellins are encoded by *flaA (swp1510)* and *lafA (swp5118)*, respectively, and their molecular weights are 47.17 KDa and 27.88 KDa, respectively, as deduced from the peptide sequences. To investigate the effect of *flgH* and *flgL* genes mutations on flagella production, filaments from the tested cell samples were extracted and flagellins were analysed by dodecyl sulphate-polyacrylamide gel electrophoresis (SDS-PAGE). We noticed that the protein band corresponding to polar flagellin was absent after *flgH1* or *flgL1* was deleted, indicating that PF cannot be synthesized in these two mutants. However, the protein band corresponding to lateral flagellin was observed simultaneously, suggesting the defect of PF synthesis induced the production of LF (Fig. 2). As expected, the polar flagellin was absent in the double deletion mutants Δ*flgH1*Δ*flgH2* and Δ*flgL1*Δ*flgL2*, and the re-introduction of the LF gene recovered the synthesis of the lateral flagellin.

**Figure 3:**
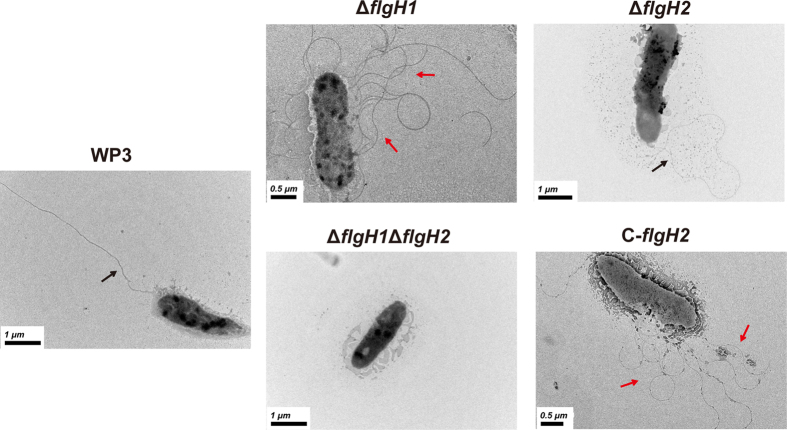
Transmission electron microscopy (TEM) analysis of WP3 flagellar gene mutants that were cultured in liquid media. The black and red arrows indicate the polar flagellum and lateral flagella, respectively. The scale bars are indicated in the lower-left corner.

Transmission electron microscopy (TEM) was used to further confirm the flagella production in different mutants. Consistent with our previous study^9^, the wild-type strain produced a single polar flagellum in liquid medium. Multiple LF and a single PF were produced in Δ*flgH1* and Δ*flgH2*, respectively. As expected, the double mutant Δ*flgH1*Δ*flgH2* was not able to produce either polar or lateral flagella, and the LF could be synthesized in the complemented strain C-*flgH2* (Fig. 3). These results indicated that the mutation of PF genes could induce the production of LF in a liquid environment.

### The disruption of LF and PF genes results in opposite changes in the transcription of flagellar genes

**Figure 4:**
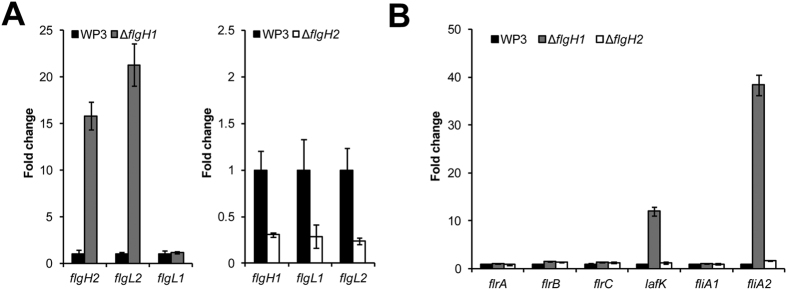
Assay of the relative transcriptional levels of flagellar structural (**A**) and regulatory (**B**) genes in WP3 flagellar gene mutants. The transcription level of these genes in WP3 was set as 1. The data shown represent the results of two independent experiments, and the error bars indicate the standard deviation of four replicates.

The swimming motility was surprisingly decreased in the LF gene deletion mutants Δ*flgH2* and Δ*flgL2*. To further explore the underlying mechanisms, qPCR was performed to quantify the relative transcriptional levels of the PF genes *flgH1* and *flgL1* between the WP3 wild-type strain and Δ*flgH2*. The results showed that the disruption of the LF gene *flgH2* significantly down-regulated the transcription of the PF genes *flgH1* and *flgL1* (Fig. 4A), which is consistent with the results of the motility assay (Fig. 1C and 1D). Additionally, the induction of LF genes in Δ*flgH1* and Δ*flgL1* was also assessed by qPCR, which showed that the LF genes *flgH2* and *flgL2* were strongly up-regulated after *flgH1* deletion (Fig. 4A).

To investigate the mechanism underlying the up- and down-regulation, the relative transcriptional levels of regulatory flagellar genes in Δ*flgH1* and Δ*flgH2* were assessed by qPCR. We noticed the mRNA level of two genes, *lafK* and *fliA2*, which encode the lateral flagellar regulator and flagellar specific sigma-28 factor, respectively, were highly increased in the PF gene mutant Δ*flgH1*, indicating that these two regulators were responsible for the up-regulation of LF gene in Δ*flgH1*. However, no significant change in the regulators *flrABC* and *fliA1*, which are involved in polar flagellar regulation, was observed, suggesting that the down-regulation of PF genes in Δ*flgH2* was achieved through an alternative mechanism. Taken together, the qPCR results demonstrated that the disruption of LF and PF genes lead to opposite changes in the transcription of flagellar genes, thus explained the results of motility assay.

### The LF gene *flgH2* can compensate for a mutation in the PF gene *flgH1*

**Figure 5:**
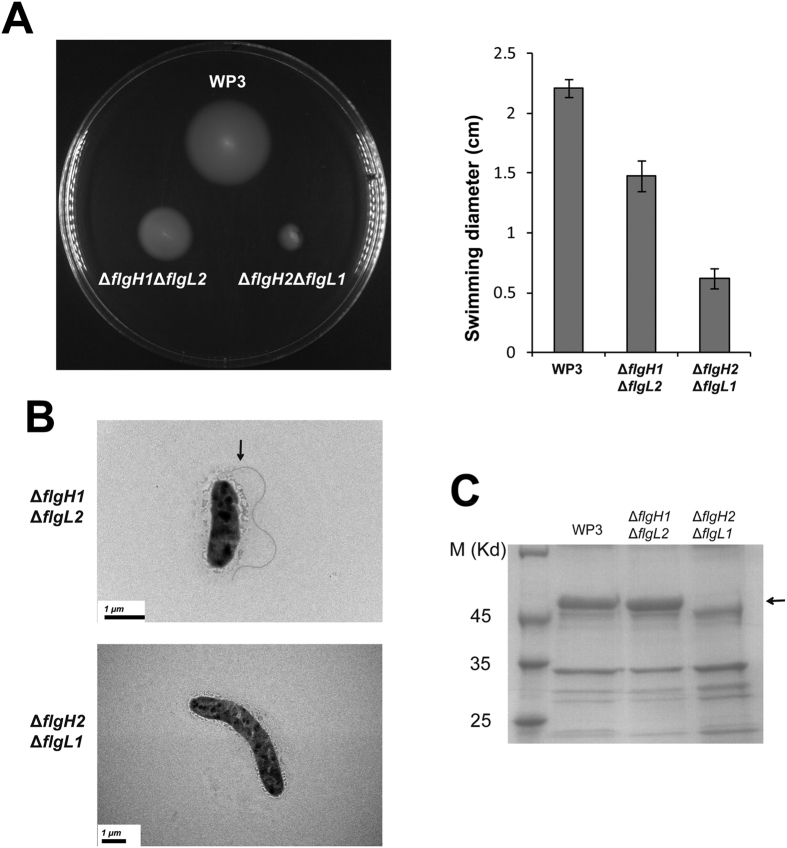
The effect of non-allelic flagellar genes mutations on swimming motility and flagellar production. (**A**) Swimming motility assays of the flagellar gene deletion mutants. The results represent at least three independent experiments and the error bars indicate the standard deviation. (**B**) TEM analysis of WP3 flagellar gene mutants that were cultured in liquid media. The black arrow indicates the polar flagellum. The scale bars are indicated in the lower-left corner. (**C**) SDS-PAGE analysis of isolated flagellins from WP3 flagellar gene mutants. Flagellins from the same volume of log-phase cultures adjusted to the same optical density were extracted and separated using SDS-PAGE. The black arrow indicates the band representing the polar flagellin. The full-length gels are presented in Supplementary Figure S5.

The compensatory activation between PF and LF regulatory genes was observed in *V. parahaemolyticus*; however, it is unclear whether PF and LF structural genes can compensate for each other. To evaluate this possibility, the two non-allelic genes *flgH1* and *flgL2* were knocked out, and the double gene deletion mutant Δ*flgH1*Δ*flgL2* was constructed. The mutant maintained a decrease in swimming motility compared to the wild-type strain (Fig. 5A). The TEM results demonstrated that a single polar flagellum was synthesized and that the polar flagellin was produced normally (Fig. 5B and 5C). These results demonstrated that the LF gene *flgH2* can compensate for the deletion of the PF gene *flgH1*. However, the PF gene *flgL1* cannot substitute for LF gene *flgL2* to produce lateral flagella, as no lateral flagella were observed.

Further, another non-allelic gene mutant Δ*flgH2*Δ*flgL1* was constructed to test whether a compensation between *flgL2* and *flgL1* could occur. However, neither PF nor LF was observed in Δ*flgH2*Δ*flgL1,* and the production of flagellin was abolished (Fig. 5B and 5C). Notably, the swimming motility is this strain was almost completely lost compared to the WP3 wild-type strain (Fig. 5A). The above results indicated that compensation between LF and PF genes may not ubiquitous in the flagellar systems.

### LF, but not PF, genes were responsible for LF synthesis and swarming motility

**Figure 6:**
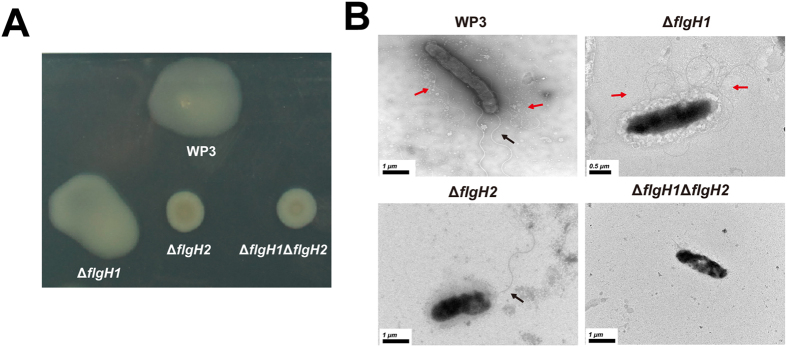
Characterization of the swarming motility and flagellar production of WP3 flagellar gene mutants on agar plates. (**A**) Swarming motility assays of the flagellar gene deletion mutants. The results represent at least three independent experiments. (**B**) TEM analysis of the WP3 flagellar gene mutants that were cultured on a swarming agar plate. The black and red arrows indicate the polar flagellum and lateral flagella, respectively. The scale bars are indicated in the lower-left corner.

The relationship between the two flagellar systems was further investigated on a solid surface, which is characterized as a habitat where LF-mediated swarming motility is important^12^. As demonstrated in Fig. 6A, the swarming motility was completely abolished in the LF single-gene mutant of WP3, Δ*flgH2*, and the double deletion mutant Δ*flgH1*Δ*flgH2*. Moreover, the PF gene deletion mutant Δ*flgH1* retained a swarming motility similar to the wild-type strain (Fig. 6A). The production of flagella was further verified by TEM observation. There was no LF synthesis after the LF genes were deleted, while the PF was still produced in PF gene mutants (Fig. 6B). Notably, the swarming motility and lateral flagella production of the non-allelic genes mutant Δ*flgH1*Δ*flgL2 and* Δ*flgH2*Δ*flgL1* were abolished (Supplementary Fig. S2). These results indicated that LF genes are specifically responsible for LF synthesis and swarming motility.

## Discussion

**Figure 7:**
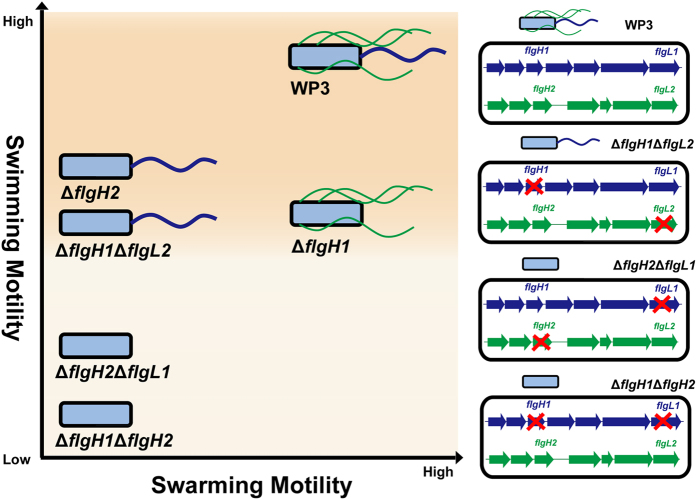
Schematic model of the relationships between the two flagellar systems in WP3. The X- and Y-axes represent the relative swarming and swimming motility, respectively. The WP3 strains with different genotype are located in the figure according to the results of motility assay and TEM observation. The polar flagellum and lateral flagella are indicated in blue and green colours, respectively. The red crosses indicate the inactivity of related flagellar genes.

Recently, increasing evidence has indicated that the distribution of the dual flagellar system is more widespread in prokaryotes than previously estimated^7^,^13^,^14^,^15^,^16^,^17^,^18^,^19^,^20^. Moreover, the origins of the secondary (lateral) flagellar system have been investigated by comparative genomic and phylogenetic analyses^21^. However, very few attempts have been conducted to investigate the relationship between genes in these two flagellar systems. In this study, the effect of induction, repression and substitution between the PF and LF genes of WP3 has been identified. The induction of LF synthesis in liquid media following the disruption of PF or a decrease in PF motor rotation rate has been demonstrated in *V. parahaemolyticus* and *Azospirillum lipoferum*^22^,^23^, and this phenomenon was also observed in WP3 (Fig. 1). Interestingly, the deletion of a WP3 LF gene significantly influenced the transcription of PF genes and swimming motility (Figs 1 and 3). To our knowledge, this is the first report of the effect of the LF system on PF function. Based on the above data, the relationship between the PF and LF genes was illustrated (Fig. 7). The disruption of the PF gene can induce the expression of LF genes and production of lateral flagella. Additionally, both the transcription of PF genes and swimming motility were decreased by the LF gene mutation. It should be noted that the level of PF flagellin remained unchanged in LF gene mutants (Fig. 2), indicating the deficiency of LF gene did not affect the expression of all PF components, and the underlying mechanism warrant further investigation.

Among the 27 sequenced genomes of *Shewanella* strains, the PF system is present in all of them, and 10 of the strains (37%) carried the dual flagellar system, suggesting that the LF system is widespread within the *Shewanella* genus (Supplementary Table S2). To investigate the origin and evolutionary history of the LF gene of WP3, a phylogenetic tree was constructed based on the conserved flagellar protein FlgH (Supplementary Table S3). The PF and LF proteins of WP3 were significantly separated and clustered with other homologous proteins that belong to the PF and LF system, respectively (Supplementary Fig. S3). Moreover, the divergence between the PF and LF proteins was identified at the order level, indicating these two systems differentiated before the formation of the *Shewanella* genus. This result was in accordance with the previous study that demonstrated that the lateral flagellar system originated first in the α-proteobacterial lineage and again in the common ancestor of the β and γ-proteobacteria^21^. Considering the widespread presence of the LF system in *Shewanellaea*, it is unlikely that it was obtained by lateral gene transfer. Interestingly, the LF proteins of two other deep-sea bacteria, *Photobacterium profundum* SS9 and *Pseudoalteromonas* sp. SM9913, were in the same branch with *Shewanella* strains, which suggests a shared origin.

Significant differences are present between the PF and LF systems. These two types of flagella have distinct structural, motor and assembly components and are powered by different motive forces^5^. Additionally, the transcriptional hierarchy of the PF and LF systems has been shown to be specific with no interconnections^15^,^24^. Although compensatory activation of PF genes by the LF regulator LafK has been demonstrated^11^, genetic experiments in *V. parahaemolyticus*, which has a dual flagellar system, suggested that there are no shared structural components among the two motility systems and that mutants unable to swarm retain swimming motility and vice versa^10^. An investigation of the dual flagellar system of *Shewanella putrefaciens* CN-32 also demonstrated that the motor components were highly specific and were unlikely to be shared between the two flagellar systems^19^. Notably, the LF gene *flgH2* can substitute for the deleted PF gene *flgH1* to synthesize an intact polar flagellum in WP3. However, another LF gene *flgL2* cannot compensate for the defective of PF gene *flgL1*. FlgH1 and FlgH2 were the same size (224 aa) and had a high identity (36.24%) shared between them, while the difference between FlgL1 (400 aa) and FlgL2 (305 aa) was more significant (the identity was 20.75%). These results indicated that the flagellar structural components in different bacteria have diverse specificities, and the compensation of LF genes for PF genes is determined by the similarity between these pairs of genes. The low specificity may contribute to the robustness of the WP3 flagellar system under different circumstances.

Bacterial motility in the sea is common and has the potential to optimize bacterial interactions with detritus, organic particles and living organisms; thus, it has important consequences for the food web, carbon storage, and nutrient cycling in the ocean^25^. A genomic analysis suggested that a dual flagellar system is most likely a common adaptation of some benthic bacteria in the sedimentary environment^20^. Notably, dual flagellar systems have been identified in several deep-sea bacteria, which have been shown to possess dramatic adaptations to the benthic habitat^7^,^9^,^20^. Therefore, the relationship between the two flagellar systems, which was revealed in this study, contributes to our understanding of microbial adaptation strategies in deep-sea environments.

## Methods

### Bacterial strains, culture conditions and growth assays

All bacterial strains and plasmids used in this study are listed in Table 1. The *Shewanella* strains were cultured in modified 2216E marine medium (2216E) (5 g/l tryptone, 1 g/l yeast extract, 0.1 g/l FePO_4_, 34 g/l NaCl) with shaking at 220 rpm at different temperatures. *E. coli* strain WM3064 was incubated in lysogeny broth (LB) medium (10 g/l tryptone, 5 g/l yeast extract, 10 g/l NaCl) at 37 °C with the addition of 50 μg/ml DL-α, ε-diaminopimelic acid (DAP). For solid medium, agar (Sangon Inc., Shanghai, China) was added at 1.5% (w/v). The antibiotic chloramphenicol (Cm) (Sigma, St. Louis, USA) was added to the medium at 25 μg/ml and 12.5 μg/ml for *E. coli* and *Shewanella* strains, respectively, when required. The growth of the WP3 strains was determined using turbidity measurements at 600 nm with 2216E medium.

### Construction of flagellar gene deletion mutants and complemented strains

In-frame deletion mutants of the genes *flgH1, flgH2, flgL1* and *flgL2* were constructed as described previously^26^. Briefly, using the construction of Δ*flgH1* as an example: First, the upstream and downstream fragments flanking both sides of the *flgH1* gene were amplified with PCR primer pairs (Supplementary Table S1). These two fragments were used as templates for a second fusion PCR, resulting in a fragment with a deletion in the *flgH1* gene. Then, the PCR product was cloned into pRE112^27^ as an *Xba* I-*Sph* I fragment, yielding pRE112-*flgH1*. This plasmid was transformed into *E. coli* WM3064 and then moved into WP3 by two-parental conjugation. The transconjugant was selected by chloramphenicol resistance in the absence of DAP, and then verified by PCR. The WP3 strain in which pRE112-*flgH1* had been inserted into the chromosome was plated on 2216E agar medium supplemented with 10% sucrose (w/v). One successful *flgH1* deletion mutant was screened and confirmed by PCR. The same strategy was used for construction of *flgH2, flgL1*and *flgL2* in-frame deletion mutants. The double deletion mutant Δ*flgH1*Δ*flgH2* was constructed either by introducing pRE112-*flgH1* into the Δ*flgH2* mutant or by introducing pRE112-*flgH2* into the Δ*flgH1* mutant. The same strategy was used for construction of Δ*flgL1*Δ*flgL2*, Δ*flgH1*Δ*flgL2* and Δ*flgH2*Δ*flgL1.* For complementation, a *Shewanella*-*E. coli* shuttle vector, pSW2, was used as previously described^28^. In brief, the *flgH2* gene, along with its native promoter region, was amplified with *Pfu* DNA polymerase (Tiangen, Beijing, China). Both the PCR product and pSW2 were digested with *Mlu* I and *Xho* I and ligated to generate pSW2-*flgH2*. The recombinant plasmid was introduced into WM3064 and then into Δ*flgH1*Δ*flgH2* by conjugation. The complemented strain C*-flgH2* was confirmed by PCR and enzymatic digestion of the re-isolated plasmid. The same strategy was used for construction of C*-flgL2.*

### RNA isolation and real-time qPCR

The WP3 strains were inoculated into 2216E medium, and the culture was harvested when the cells reached mid-exponential phase (OD_600_ ≈ 0.6). Total RNA extraction, reverse transcription and real-time qPCR were performed as previously described^29^,^30^. The relative mRNA level of WP3 flagellar genes was quantified by the ΔΔC_T_ method^31^. The amount of target was normalized to that of the reference gene *swp2079*^32^, which has stable expression under various conditions relative to the calibrator (the transcription levels of the genes in WP3 wild-type strain were set as 1). The primer pairs (Table S1) used to amplify the selected genes via qPCR were designed using Primer Express software (Applied Biosystems, CA, USA).

### Motility assay and transmission electron microscopy (TEM)

For motility assays, 1 μl of culture (Mid-exponential phase) from each strain was placed on swimming plates (2216E medium with 0.3% agar (w/v), Eiken Chemical, Tokyo, Japan) or swarming plates (2216E medium with 0.7% agar (w/v)). For the swimming and swarming motility assays, the plates were incubated at 20 °C for 3 days and 4 days, respectively. The motility was assessed by measuring the diameter of the colony. The assays were performed at least three independent times and in triplicate. For transmission electron microscopy (TEM), bacteria were grown on swimming or swarming agar plates and suspended in 1% (w/v) sterile NaCl solution. The samples were placed onto a carbon-coated grid (200 mesh) and air-dried. The grid was then examined using a Tecnai G2 BioTwin microscope (FEI Company, Eindhoven, Netherlands).

### Extraction and analysis of flagellin

WP3 strains were grown in a 200-ml bacterial batch culture with 2216E medium, when their OD (600 nm) reached 2.0, the cultures were collected by centrifugation at 3,000 × g for 10 min at 4 °C. The cell pellet was resuspended in 5 ml sterile NaCl solution (3.4%, w/v), pH 7.0, and vortexed for 20 min to shear off flagella. The cells were removed by centrifugation at 10,000 × g for 30 min at 4 °C, and the supernatant containing flagella was filtered through a 0.45 μm-pore filter. The filtrate was incubated with polyethylene glycol 6000 (1.3% w/v) and 166 mM NaCl for 2 h at 4 °C. This suspension was centrifuged at 12,000 × g for 40 min at 4 °C, and the pellet containing purified flagella was resuspended in 100 μl phosphate-buffered saline (PBS), pH 7.0. For analysis, the samples were boiled for 10 min and then separated by sodium dodecyl sulphate polyacrylamide (12% w/v) gel electrophoresis (SDS-PAGE). The gel was stained with Coomassie brilliant blue R-250 (Sangon Inc., Shanghai, China).

## Additional Information

**How to cite this article**: Jian, H. *et al*. Characterization of the relationship between polar and lateral flagellar structural genes in the deep-sea bacterium *Shewanella piezotolerans* WP3. *Sci. Rep.* **6**, 39758; doi: 10.1038/srep39758 (2016).

**Publisher's note:** Springer Nature remains neutral with regard to jurisdictional claims in published maps and institutional affiliations.

## Supplementary Material

Supplementary Information

## Figures and Tables

**Table 1:** Bacterial strains and plasmids used in this study.

Strain or plasmid	Relevant genotype	Reference or source
*E. coli*		
WM3064	Donor strain for conjugation; Δ*dapA*	31
*S. piezotolerans* WP3		
WP3	Wild-type, GenBank accession number CP000472	Lab stock
Δ*flgH1*	WP3, *flgH1* gene deletion mutant	This work
Δ*flgH2*	WP3, *flgH2* gene deletion mutant	This work
Δ*flgH1*Δ*flgH2*	WP3, *flgH1* and *flgH2* gene double deletion mutant	This work
Δ*flgL1*	WP3, *flgL1* gene deletion mutant	This work
Δ*flgL2*	WP3, *flgL2* gene deletion mutant	This work
Δ*flgL1*Δ*flgL2*	WP3, *flgL1* and *flgL2* gene double deletion mutant	This work
C-*flgH2*	Δ*flgH1*Δ*flgH2* with pSW2-*flgH2*	This work
C-*flgL2*	Δ*flgL1*Δ*flgL2* with pSW2-*flgL2*	This work
Δ*flgH1*Δ*flgL2*	WP3, *flgH1* and *flgL2* gene double deletion mutant	This work
Δ*flgH2*Δ*flgL1*	WP3, *flgH2* and *flgL1* gene double deletion mutant	This work
Plasmids		
pRE112	Allelic-exchange vector; Cm^r^ *sacB*	27
pSW2	Shuttle vector, used for complementation; Chl^r^	32
pRE112-*flgH1*	pRE112 containing the PCR fragment for deletion of the *flgH1* gene	This work
pRE112-*flgH2*	pRE112 containing the PCR fragment for deletion of the *flgH2* gene	This work
pRE112-*flgL1*	pRE112 containing the PCR fragment for deletion of the *flgL1* gene	This work
pRE112-*flgL2*	pRE112 containing the PCR fragment for deletion of the *flgL2* gene	This work
pSW2-*flgH2*	pSW2 containing the *flgH2* gene for complementation	This work
pSW2-*flgL2*	pSW2 containing the *flgL2* gene for complementation	This work
