# Supplementary material for: Characterization of the relationship between polar and lateral flagellar structural genes in the deep-sea bacterium Shewanella piezotolerans WP3
Source: Sci Rep. 2016 Dec 22;6:39758. doi: 10.1038/srep39758 (PMC5178100; doi:10.1038/srep39758)
Supplement: Supplementary Information [file srep39758-s1.pdf]

Supplementary Information

Characterization of the relationship between polar and lateral flagellar structural genes in the deep-sea bacterium *Shewanella piezotolerans* WP3

Huahua Jian[1], Han Wang[1], Xianping Zeng[1], Lei Xiong[1], Fengping Wang[1], Xiang Xiao[1,2*]

1, State Key Laboratory of Microbial Metabolism, School of Life Sciences and Biotechnology, Shanghai Jiao Tong University, Shanghai, PR China

2, State Key Laboratory of Ocean Engineering, School of Naval Architecture, Ocean and Civil Engineering, Shanghai Jiao Tong University, Shanghai, PR China

* Correspondence to Xiang Xiao, xoxiang@sjtu.edu.cn

Figures

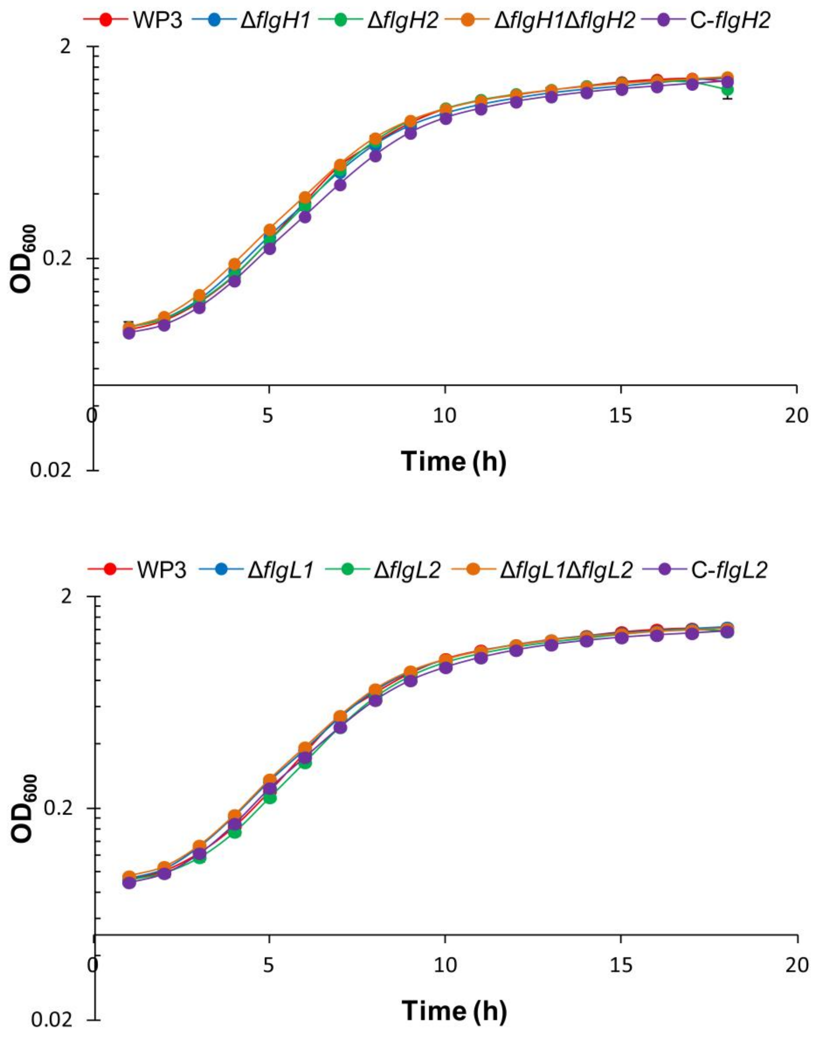

Figure S1. Growth assay of WP3 flagellar gene deletion mutants. The growth of WP3 and the mutants was monitored by measuring the optical density of the cultures at OD_{600}. The mean values with standard deviations (indicated by vertical bars) from triplicate experiments are given.

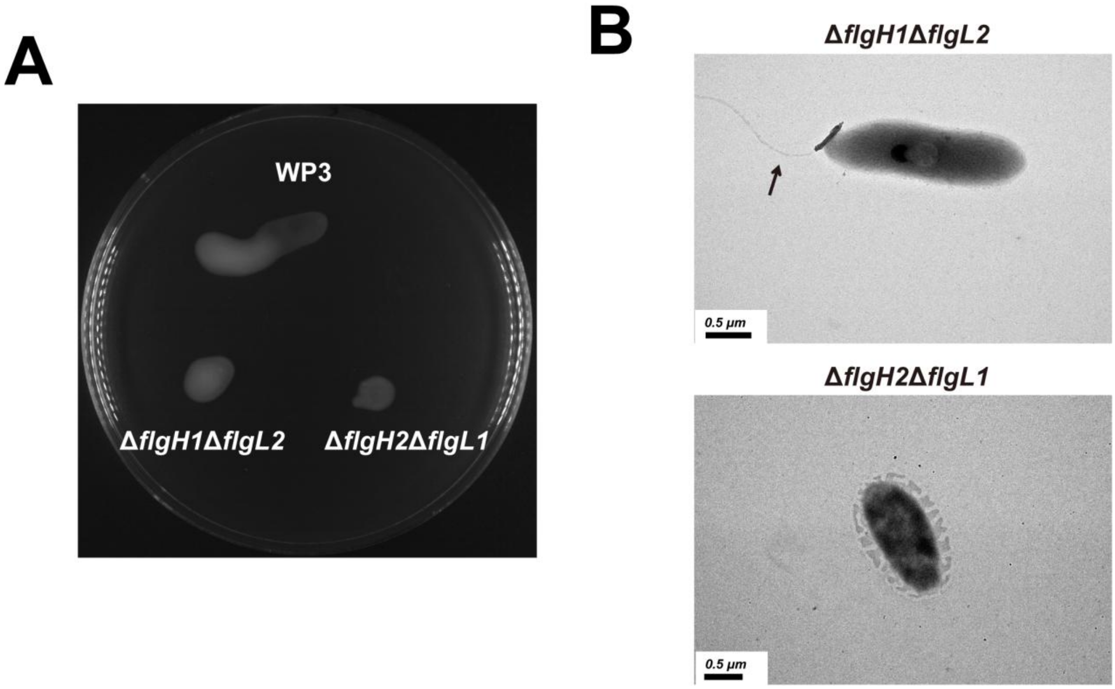

Figure S2. Effect of non-allelic flagellar genes mutation on the swarming motility and flagellar production on agar plates. (A) Swarming motility assays of the flagellar gene deletion mutants. The results represent two independent experiments. (B) TEM analysis of WP3 flagellar gene mutants that were cultured on swarming agar plate. Black arrow indicates the polar flagellum. The scale bars are indicated in the lower-left corner.

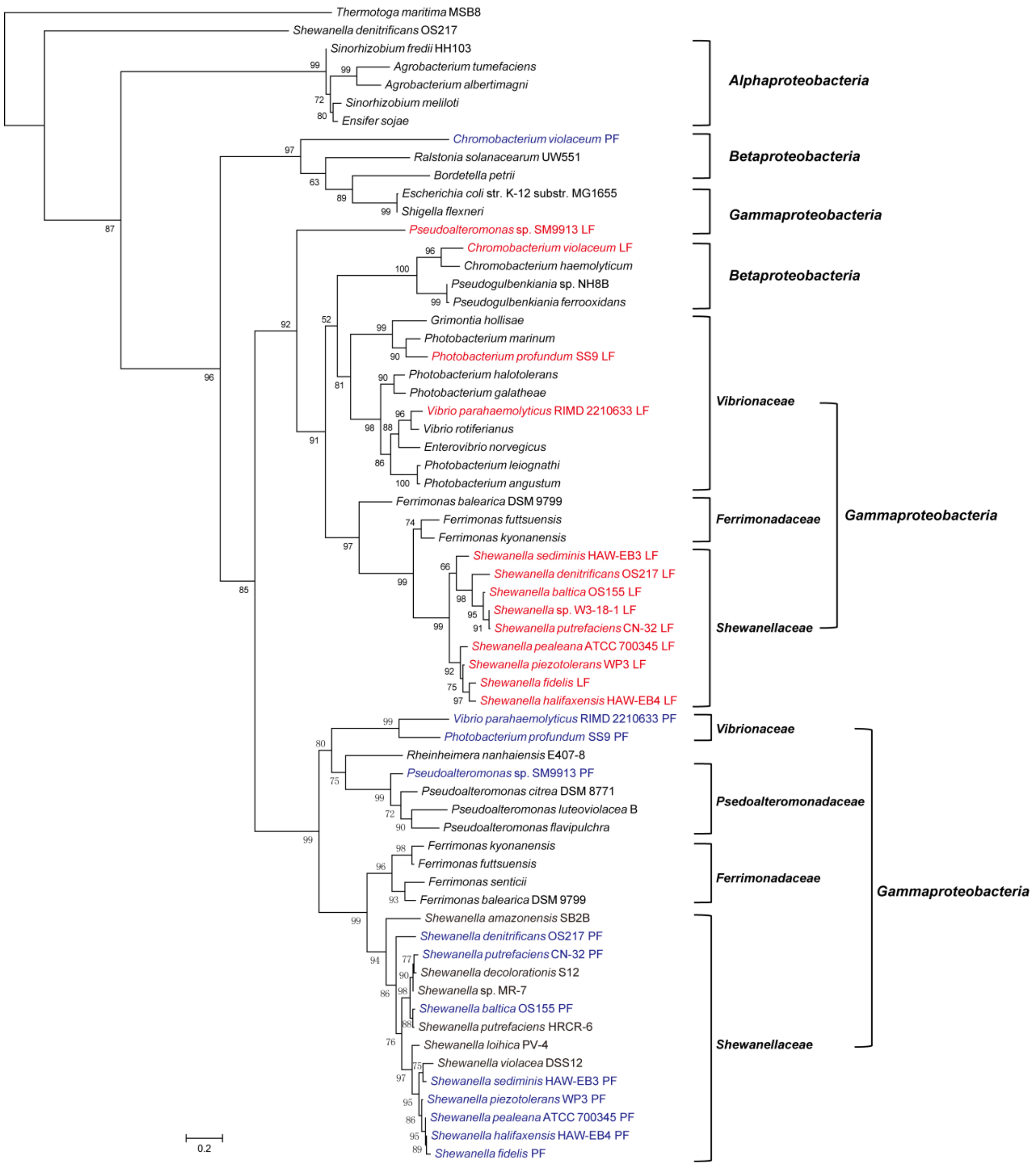

Figure S3. Phylogenetic tree of FlgH protein sequences of proteobacteria. "PF" and "LF" after species names indicate polar and lateral flagellar systems, which are shown in blue and red, respectively. FlgH of *Thermotoga maritima* was used as the out-group. The scale bar represents amino acid substitutions per site, and numbers represent the branch support values in percentage. For each data set, bootstrap values were obtained with 1,000 replicates.

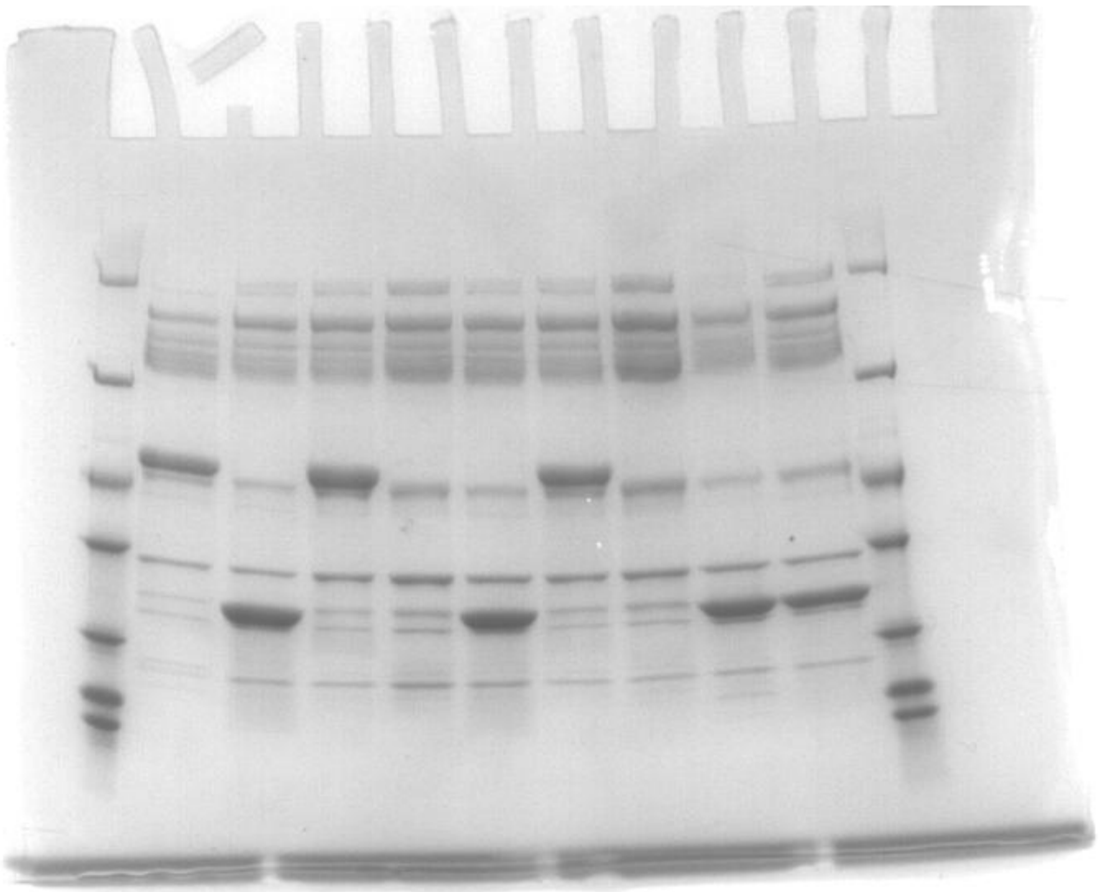

Figure S4. SDS-PAGE analysis of isolated flagellins from WP3 flagellar gene mutants and complemented strains.

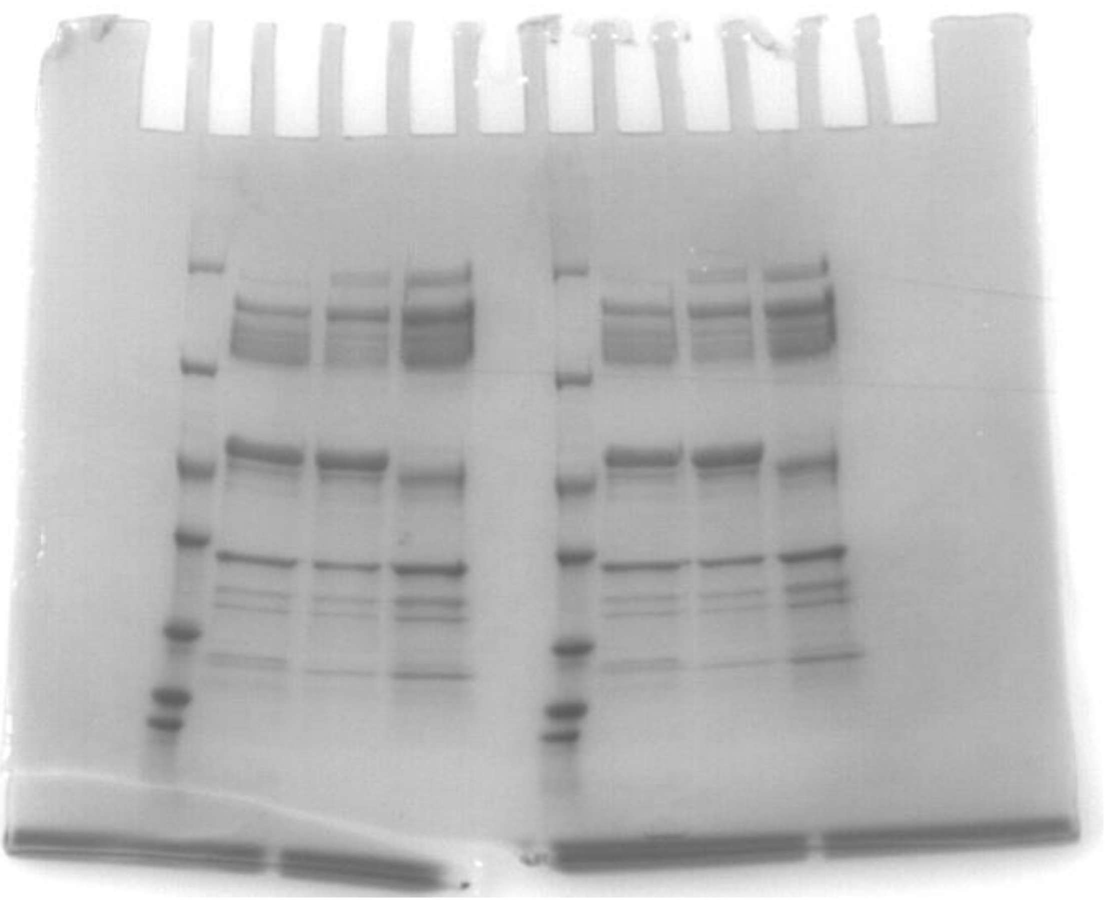

Figure S5. SDS-PAGE analysis of isolated flagellins from WP3 flagellar gene mutants.

Tables

Table S1. Primers and probe used in this study

Primer name	Sequence (5’-3’)	Description
**Swp1504UL**	TATCTAGACAAGCGGGTGGGGTAAGTG	*flgH1* deletion
**Swp1504UR**	CGCTATATAGGATAAACAGGCGCATAATAAGG	*flgH1* deletion
**Swp1504DL**	TTTATCCTATATAGCGGCACTGGAACATTTG	*flgH1* deletion
**Swp1504DR**	TAGCATGCGGCGGCATATTGGCACTCACT	*flgH1* deletion
**Swp5111UL**	ATGAGCTCAGGTCAAGCCGATGCCCAAGAG	*flgH2* deletion
**Swp5111UR**	CGGATAAGCAAGCATCGCGATTCCAAGTCCTA	*flgH2* deletion
**Swp5111DL**	GATGCTTGCTTATCCGCGGCGAAAAATGG	*flgH2* deletion
**Swp5111DR**	TTTCTAGATTAATGCGCTGGGAGGAGATGTCA	*flgH2* deletion
**Swp1508UL**	AAGAGCTCGCGGCAAACTCGGGCAACA	*flgL1* deletion
**Swp1508UR**	ATTGATTGACCATCGCAGACTGGCTTTTCATCACAT	*flgL1* deletion
**Swp1508DL**	GCGATGGTCAATCAATCGGCACTGTCTT	*flgL1* deletion
**Swp1508DR**	AAGGTACCTCTGTATCACCATTGCCGCTGTTC	*flgL1* deletion
**Swp5115UL**	ATGAGCTCGCGGGAACAACAGCATCAAAAAT	*flgL2* deletion
**Swp5115UR**	TTGAGTGCATCATCAGAGGGGCGCAGTATCG	*flgL2* deletion
**Swp5115DL**	CTGATGATGCACTCAAGGTCACCCAGCAAACA	*flgL*2 deletion
**Swp5115DR**	ATTCTAGAAGCGGGCAATAAGACTCGGGAACT	*flgL2* deletion
**C-Swp5111For**	GACTGGTACCTAATTGACTTGAAAAGGATC	*flgH2* complementation
**C-Swp5111Rev**	ACGGACGCGTAGTGATTAAACTTCATTGGA	*flgH2* complementation
**C-Swp5115For**	TGTAGGTACCATCCTACCAGGCTAACGC	*flgL2* complementation
**C-Swp5115Rev**	TCTACTGCAGATCAGTCAGTGGCACCTTA	*flgL2* complementation
**Swp1504RTFor**	CCATTGATTCACCGCGAGTA	qPCR
**Swp1504RTRev**	CAAATGTTCCAGTGCCGCTAT	qPCR
**Swp1508RTFor**	GATGCTGCGCTCAGTAAACG	qPCR
**Swp1508RTRev**	TCTCATCGGCAATTGTTTGC	qPCR
**Swp5111RTFor**	TTTGATCCGCACCGATGAT	qPCR
**Swp5111RTRev**	GCGATGCGCTGAGAAGAGA	qPCR
**Swp5115RTFor**	AGCCGCGCCGAAACTTAT	qPCR
**Swp5115RTRev**	TCTCGCATACGCGTTTGC	qPCR
**Swp2079RTFor**	TTAAGGCAATGGAAGCTGCAT	qPCR
**Swp2079RTRev**	CGTCTTTACCCGTTAATGATACGA	qPCR
**Swp1538RTFor**	GTCGCGGTCGATGTGGTT	qPCR
**Swp1538RTRev**	GCGACTCATAAGCAACATCATCA	qPCR
**Swp5124RTFor**	TTTTGGCCATCGAAGACATG	qPCR
**Swp5124RTRev**	GCCTTGCGGACTCGAGTAAC	qPCR
**Swp1516RTFor**	TCCGGTTAACTGTGGTGCAA	qPCR
**Swp1516RTRev**	TGGCCGAATAGCTCACTCTCA	qPCR
**Swp1517RTFor**	CAATAGCGGCACAAAAAAGCT	qPCR
**Swp1517RTRev**	CGAATACACTGTGTCGCATGATT	qPCR
**Swp1518RTFor**	ACGACAGCTTTGACATGGTGAT	qPCR
**Swp1518RTRev**	CCAAGGCCACCAATTCCTT	qPCR

**Swp5090RTFor**	TCGCTGTTGCATCGATTACG	qPCR
**Swp5090RTRev**	AGCGCTGACGAGATCGTTTT	qPCR

Table S2. The sequenced *Shewanella* strains and their flagellar system

No	Species or strain	Geographical origin	Isolation-site characteristics	Genome (Mb)	Flagellar system
**1**	***Shewanella amazonensis* SB2B**	Amapa River, Brazil	Sediment; suboxic redox conditions; 1 m	4.31	PF
2	***Shewanella loihica* PV-4**	Hawaiian Sea mount, United States	Iron-rich mat; hydrothermal vent; 1,325 m	4.6	PF
3	***Shewanella piezotolerans* WP3**	West Pacific site WP (142°E, 8°N)	Sediment; under 1,914 m of water	5.4	PF+LF
4	***Shewanella pealeana* ATCC 700345**	Woods Hole Harbor, Massachusetts, United States	Squid nidamental gland	5.17	PF+LF
**5**	***Shewanella halifaxensis* HAW-EB4**	Halifax Harbor, Nova Scotia, Canada	Sediment; munitions dumping area; 215 m, Atlantic Ocean	5.23	PF+LF
6	***Shewanella psychrophila* WP2**	West Pacific site WP (142°E, 8°N)	Sediment; under 1,914 m of water	6.35	PF+LF
**7**	***Shewanella violacea* DSS12**	Ryuku Trench, Philippine Sea	Sediment; 5,110 m	4.96	PF
**8**	***Shewanella benthica* KT99**	Tonga-Kermadec Trench	Deep sea; high-pressure zone; 9,000 m	4.35	PF
**9**	***Shewanella sediminis* HAW-EB3**	Halifax Harbor, Nova Scotia, Canada	Sediment; 50 nautical miles from shore; 215 m	5.52	PF+LF
**10**	***Shewanella woodyi* ATCC 51908 *(*MS32)**	Strait of Gibraltar, Mediterranean Sea	Detritus; 370 m	5.94	PF
**11**	***Shewanella denitrificans* OS217**	Baltic Sea	Sea-water; oxic–anoxic interface; 120 m	4.55	PF+LF
**12**	***Shewanella frigidimarina* NCIMB 400**	Coast of Aberdeen, United Kingdom	Sea-water; North Sea	4.85	PF
**13**	***Shewanella putrefaciens* 200**	Alberta, Canada	Crude-oil pipeline	4.84	PF
**14**	***Shewanella putrefaciens* CN-32**	Albuquerque, New Mexico, United States	Subsurface; shale sandstone; 250 m	4.66	PF+LF
15	***Shewanella* sp. W3-18-1**	Washington coast, Pacific Ocean	Marine sediment; under 997 m of oxic water	4.71	PF+LF
**16**	***Shewanella baltica* OS223**	Baltic Sea	Sea-water; anoxic zone; 140 m	5.15	PF
**17**	***Shewanella baltica* BA175**	Baltic Sea	ND	5.20	PF
**18**	***Shewanella baltica* OS117**	Baltic Sea	ND	5.53	PF
**19**	***Shewanella baltica* OS155**	Baltic Sea	Sea-water; oxic zone; 2 ml per litre of oxygen; 90 m	5.34	PF+LF
**20**	***Shewanella baltica***	Baltic Sea	Sea-water; oxic–anoxic interface;	5.31	PF

	**OS185**		120 m		
**21**	***Shewanella baltica* OS678**	Baltic Sea	low-oxygen zone just above the chemocline (110 m)	5.37	PF
**22**	***Shewanella baltica* OS195**	Baltic Sea	Sea-water; anoxic zone; 140 m	5.55	PF
**23**	***Shewanella oneidensis* MR-1**	Lake Oneida, New York, United States	Sediment; anaerobic; Mn(IV) reduction	5.13	PF
**24**	***Shewanella* sp. ANA-3**	Woods Hole, Massachusetts, United States	Brackish water; arsenic-treated wooden pier	5.25	PF
**25**	***Shewanella* sp. MR-7**	Black Sea	Sea-water; anoxic zone; high NO_3; 60 m	4.79	PF
**26**	***Shewanella* sp. MR-4**	Black Sea	Sea-water; oxic zone; 16℃; 5 m	4.71	PF
**27**	***Shewanella fidelis* ATCC BAA-318**	The South China Sea	Sediment; depth of 73 m	4.8	PF+LF

Table S3. The protein sequence of FlgH used in the pylogenetic analysis

No	Class	Species	gi Number
1	***Thermotogae***	*Thermotoga maritima* MSB8	15644288
2	***Gammaproteobacteria***	*Shewanella denitrificans* OS217	91713419
3	***Alphaproteobacteria***	*Sinorhizobium fredii* HH103	365177968
4	***Alphaproteobacteria***	*Agrobacterium tumefaciens*	1086565
5	***Alphaproteobacteria***	*Agrobacterium albertimagni*	493777918
6	***Alphaproteobacteria***	*Sinorhizobium meliloti*	489625448
7	***Alphaproteobacteria***	*Ensifer sojae*	736851944
8	***Betaproteobacteria***	*Chromobacterium violaceum*	768336605
9	***Betaproteobacteria***	*Ralstonia solanacearum* UW551	83725703
10	***Betaproteobacteria***	*Bordetella petrii*	754361705
11	***Gammaproteobacteria***	*Escherichia coli* str. K-12 substr. MG1655	16129042
12	***Gammaproteobacteria***	*Shigella flexneri*	745761840
13	***Gammaproteobacteria***	*Vibrio parahaemolyticus* RIMD 2210633	28805768
14	***Gammaproteobacteria***	*Photobacterium profundum*	499536872
15	***Gammaproteobacteria***	*Rheinheimera nanhaiensis* E407-8	383704066
16	***Gammaproteobacteria***	*Pseudoalteromonas* sp. SM9913	315015864
17	***Gammaproteobacteria***	*Pseudoalteromonas citrea* DSM 8771	540253670
18	***Gammaproteobacteria***	*Pseudoalteromonas luteoviolacea* B ATCC 29581	441749577
19	***Gammaproteobacteria***	*Pseudoalteromonas flavipulchra*	498292150
20	***Gammaproteobacteria***	*Ferrimonas kyonanensis*	654652128
21	***Gammaproteobacteria***	*Ferrimonas futtsuensis*	654649162
22	***Gammaproteobacteria***	*Ferrimonas senticii*	737406071
23	***Gammaproteobacteria***	*Ferrimonas balearica* DSM 9799	307632559
24	***Gammaproteobacteria***	*Shewanella amazonensis* SB2B	119767947
25	***Gammaproteobacteria***	*Shewanella denitrificans* OS217	91714669
26	***Gammaproteobacteria***	*Shewanella putrefaciens* CN-32	145565377
27	***Gammaproteobacteria***	*Shewanella decolorationis* S12	553947544
28	***Gammaproteobacteria***	*Shewanella* sp. MR-7	113888283
29	***Gammaproteobacteria***	*Shewanella baltica* OS155	125998348
30	***Gammaproteobacteria***	*Shewanella putrefaciens*	655354729
31	***Gammaproteobacteria***	*Shewanella loihica* PV-4	126637577
32	***Gammaproteobacteria***	*Shewanella violacea* DSS12	293326657
33	***Gammaproteobacteria***	*Shewanella sediminis* HAW-EB3	157318587
34	***Gammaproteobacteria***	*Shewanella piezotolerans* WP3	212555835
35	***Gammaproteobacteria***	*Shewanella pealeana* ATCC 700345	157846174
36	***Gammaproteobacteria***	*Shewanella fidelis*	655362488
37	***Gammaproteobacteria***	*Shewanella halifaxensis* HAW-EB4	167353386
38	***Gammaproteobacteria***	*Pseudoalteromonas* sp. SM9913	315014501
39	***Gammaproteobacteria***	*Ferrimonas balearica* DSM 9799	307632211
40	***Gammaproteobacteria***	*Ferrimonas futtsuensis*	654647905
41	***Gammaproteobacteria***	*Ferrimonas kyonanensis*	654653971
42	***Gammaproteobacteria***	*Shewanella sediminis* HAW-EB3	157315578

**43**	***Gammaproteobacteria***	*Shewanella denitrificans* OS217	91716994
**44**	***Gammaproteobacteria***	*Shewanella baltica* OS155	125999358
**45**	***Gammaproteobacteria***	*Shewanella* sp. W3-18-1	120557401
**46**	***Gammaproteobacteria***	*Shewanella putrefaciens* CN-32	145566237
**47**	***Gammaproteobacteria***	*Shewanella pealeana* ATCC 700345	157844899
**48**	***Gammaproteobacteria***	*Shewanella piezotolerans* WP3	212559272
**49**	***Gammaproteobacteria***	*Shewanella fidelis*	655361441
**50**	***Gammaproteobacteria***	*Shewanella halifaxensis* HAW-EB4	167356180
**51**	***Gammaproteobacteria***	*Chromobacterium violaceum*	768336826
**52**	***Gammaproteobacteria***	*Chromobacterium haemolyticum*	759908292
**53**	***Gammaproteobacteria***	*Pseudogulbenkiania* sp. NH8B	503853881
**54**	***Gammaproteobacteria***	*Pseudogulbenkiania ferrooxidans*	496241021
**55**	***Gammaproteobacteria***	*Grimontia hollisae*	750224026
**56**	***Gammaproteobacteria***	*Photobacterium marinum*	494727063
**57**	***Gammaproteobacteria***	*Photobacterium profundum*	752746676
**58**	***Gammaproteobacteria***	*Photobacterium halotolerans*	653000184
**59**	***Gammaproteobacteria***	*Photobacterium galatheae*	636574688
**60**	***Gammaproteobacteria***	*Photobacterium angustum*	767119450
**61**	***Gammaproteobacteria***	*Photobacterium leiognath*	767076517
**62**	***Gammaproteobacteria***	*Enterovibrio norvegicus*	515572155
**63**	***Gammaproteobacteria***	*Vibrio rotiferianus*	657850858
**64**	***Gammaproteobacteria***	*Vibrio parahaemolyticus* RIMD 2210633	28900125
